# Automatic Methodology for Forest Fire Mapping with SuperDove Imagery

**DOI:** 10.3390/s24165084

**Published:** 2024-08-06

**Authors:** Dionisio Rodríguez-Esparragón, Paolo Gamba, Javier Marcello

**Affiliations:** 1 Instituto de Oceanografía y Cambio Global, IOCAG, Unidad Asociada ULPGC-CSIC, 35017 Las Palmas de Gran Canaria, Spain; javier.marcello@ulpgc.es; 2 Department of Electrical, Biomedical and Computer Engineering, University of Pavia, 27100 Pavia, Italy; paolo.gamba@unipv.it

**Keywords:** burned-area mapping, severity mapping, wildfire, SuperDove, PlanetScope, vegetation index, k-means, global warning, climate change

## Abstract

The global increase in wildfires due to climate change highlights the need for accurate wildfire mapping. This study performs a proof of concept on the usefulness of SuperDove imagery for wildfire mapping. To address this topic, we present an automatic methodology that combines the use of various vegetation indices with clustering algorithms (bisecting k-means and k-means) to analyze images before and after fires, with the aim of improving the precision of the burned area and severity assessments. The results demonstrate the potential of using this PlanetScope sensor, showing that the methodology effectively delineates burned areas and classifies them by severity level, in comparison with data from the Copernicus Emergency Management Service (CEMS). Thus, the potential of the SuperDove satellite sensor constellation for fire monitoring is highlighted, despite its limitations regarding radiometric distortion and the absence of Short-Wave Infrared (SWIR) bands, suggesting that the methodology could contribute to better fire management strategies.

## 1. Introduction

Global warming, and the consequent long-term increase in the Earth’s average temperature, has been a topic of concern for citizens, scientists, and policy makers in recent decades [1]. One of the most alarming consequences of this warming trend is the increase in the incidence and intensity of fires, especially forest fires [2,3,4].

Forest fires, also known as wildfires, have been a natural part of the Earth’s ecology for millions of years [5]. However, recent data suggest a worrying trend: the frequency, intensity, and total area burned by these fires have increased at an unprecedented rate. This escalation is not limited to just one or two regions; it is a global phenomenon that affects forests from the Amazon and Australia to California and Siberia [6,7,8,9].

The connection between global warming and increased fire activity is complex but undeniable. Warmer temperatures lead to more frequent and longer droughts, creating drier conditions that are ideal for fires to ignite and spread. Additionally, warmer weather has led to earlier snowmelt and, consequently, a longer fire season [3,10].

Although, as mentioned, wildfires are a natural and integral component of many ecosystems, they have significant impacts on the environment, the economy, and human health [11,12]. This is why the ability to accurately estimate the burned area of these fires is crucial for several reasons. Wildfires can dramatically alter the landscape and disrupt ecosystems [13]. They can lead to a loss of vegetation, changes to wildlife habitats, and increased vulnerability to erosion and landslides [14]. The accurate estimation of burned areas allows for a better understanding of these environmental impacts and assists in the development of effective post-fire recovery strategies [15]. Similarly, wildfires contribute to climate change by releasing large amounts of carbon dioxide and other greenhouse gases into the atmosphere. Therefore, an accurate estimate of the burned area is also required to quantify these emissions and improve the accuracy of climate models. Additionally, it is vital for effective fire management [16]. An accurate estimate helps evaluate the effectiveness of firefighting efforts, plan future resource allocation, and develop strategies to prevent or mitigate the impact of future fires [17,18]. Finally, wildfires can cause substantial economic damage, including property loss and costs associated with firefighting efforts. They also pose serious health risks due to the release of harmful contaminants [19]. Generating detailed burned-area maps can help assess these health and economic impacts, inform policy decisions, and guide public health responses.

The most common methodologies for determining the burned area and severity of fires using remote sensing images are based on the analysis of time series of multispectral images, statistical modeling, and unsupervised classification [20]. In particular, spectral indices are commonly considered, with the Normalized Burned Area Index (NBR) standing out as the most used, which is calculated from Near-Infrared (NIR) and Short-Wave Infrared (SWIR) bands. Determining the burned area and fire severity is usually estimated by comparing images from before and after the fire [21,22].

CubeSats, such as those in the PlanetScope constellation (Planet Labs Inc., San Francisco, CA, USA, © 2023 Planet Labs PBC), offer an opportunity to attain better spatial and temporal resolution to help address this problem more efficiently, despite the absence of the SWIR band. These satellites provide high-resolution images with a very short revisit time, allowing more frequent monitoring of areas affected by fires [23,24,25,26]. Although the lack of the SWIR band, which is useful in distinguishing burned areas, can be a challenge, several studies have shown that accurate results can be obtained using optimal band combinations or deep learning techniques [27,28,29,30,31]. Additionally, CubeSats’ ability to image small areas at high frequency makes them especially useful for monitoring fires on islands and other small areas that may be difficult to monitor with other satellites.

The growing interest in the potential of the PlanetScope constellation has sparked the interest of the scientific community. Thus, in [32], the authors examine the effectiveness of each of the eight bands available in PlanetScope images using a variety of feature selection methods, and then use these bands to map the extent of biomass burning and consumption for three forest fires. Their approach includes supervised classification methods, such as support vector machine and principal component analysis. In [27], a burned area mapping method to extract damaged areas using deep learning (DL)-based semantic segmentation, specifically, a U-Net that requires a unitemporal PlanetScope image, is presented. From another point of view, a framework for unattended wildfire damage assessment using VHR satellite imagery with PlanetScope data is proposed in [33]. The approach of these authors aims to take advantage of the high revisit frequency of this sensor. Alternatively, the authors in [34] use images from the PlanetScope sensor to generate high-resolution fire perimeters as a Sentinel-2 fire reference for the evaluation of burned-area products in Latin America and the Caribbean for the year 2019. Finally, a combination of DL Transformer methods and high-resolution PlanetScope images to map burned areas in the Brazilian Pantanal wetland is introduced in [35].

In this study, a proof of concept is presented for the detection of burned areas and the assessment of the severity of fires using different vegetation indices and the images of the SuperDove sensor of the PlanetScope constellation. To this aim, a methodology is developed that is then specifically applied to four fires in different areas of Greece and Spain. SuperDove’s high-resolution imagery enables the detailed monitoring of wildfires, providing a unique opportunity to improve our understanding of these devastating events and develop more effective management strategies. Through this work, we seek to contribute to the existing literature on wildfire detection and severity assessment and demonstrate the usefulness of SuperDove imagery in this context. To the best of our knowledge, this contribution differs from previous studies by offering a robust approach to delineate burned areas and classify them according to severity level, especially for smaller fires, which is essential to understand the evolution of fire–climate relationships in the current world setting. It is also different from other methods that rely on fixed thresholds or supervised classifications, providing a flexible and potentially more accurate alternative.

## 2. Materials and Methods

This section details the materials and methods used in this study. It includes a description of the images of burned areas and the ancillary data required for the analysis. Additionally, the vegetation indices tested on the SuperDove images are presented. Finally, the methodology employed to synthesize both burned-area and severity maps is outlined.

### 2.1. Materials

To evaluate the performance of the Planetscope sensor images for the analysis of burned areas and fire severity, a specific methodology was applied to four selected areas affected by fire, namely, three events in August 2023 (Parnitha Mountain and Euboea in Greece, and Portbou in Spain), as well as one in September 2022 (Sierra de los Guájares in Spain).

- Parnitha Mountain is located north of Attica in Greece. It is an area of great ecological and cultural importance for Greece due to its rich biodiversity, water resources, historical value, and recreational potential that make it an invaluable asset to the region. A fire started on 22 August 2023, near the Kleiston monastery in that area, causing the destruction of houses and vehicles in the suburb of Fyli. The impact of the fire was significant: it burned more than 6000 hectares of land and potentially affected many buildings [36].
- Portbou is in the Alto Ampurdán region, in the province of Gerona, Catalonia, Spain. It is a town on the border with France, located on the Mediterranean coast, about 10 km from the French border. A wildfire hit the area on 4 August 2023. The significance of the fire arises from its location in a border city near residential areas, threatening both natural habitats and human populations. The fire destroyed about 500 hectares, mainly of vegetation cover [37].
- The island of Euboea is in Greece, off the eastern coast of the Aegean Sea. It has great importance due to its diverse natural resources, its tourist attractions, and its rich biodiversity. In this sense, it is a habitat for many birds, reptiles, and mammals, including some endangered species. A fire started on 21 August 2023 and spread widely, covering a wide front that threatened valuable forests and farmland. The fire burned an area of more than 500 hectares [38].
- Sierra de Los Guajares is in the province of Granada in southern Spain. It is an area of environmental importance that has a notable variety of plant and animal life, many of which are threatened. The area is of great economic interest due to its use for farming and hunting, and its cultural heritage. A devastating wildfire swept through the region in September 2022, burning more than 5000 hectares of land, mostly forests [39].

The fires in all of these areas required the activation of the Copernicus Emergency Management Service (CEMS) for comprehensive analysis and support [40]. The CEMS is part of the EU Copernicus program and provides satellite-based geospatial information for disaster response and management. It offers rapid mapping for immediate response to different crises, including fire, as well as risk and recovery mapping for the planning and recovery phases.

To evaluate the impact of the forest fires, a meticulous process was carried out to select images for each affected area. Two images of each study area from the SuperDove sensor in the PlanetScope constellation were chosen through detailed visual inspection. Each pair for an area comprised an image from before and another after the fire. This approach allowed for a direct and effective comparison of the conditions of the area before and after the disaster. Additionally, to ensure consistency and accuracy in damage assessment, all images were downloaded with the same settings. The downloaded products corresponded to the so-called level 3B. This is an 8-band surface reflectance product that is atmospherically corrected and orthorectified, with a spatial resolution of 3 m [41]. This ensured that any differences observed between the pre- and post-fire images were due solely to the effects of the fire, thus eliminating any variables that could be attributed to changes in imaging settings.

**Figure 1:**
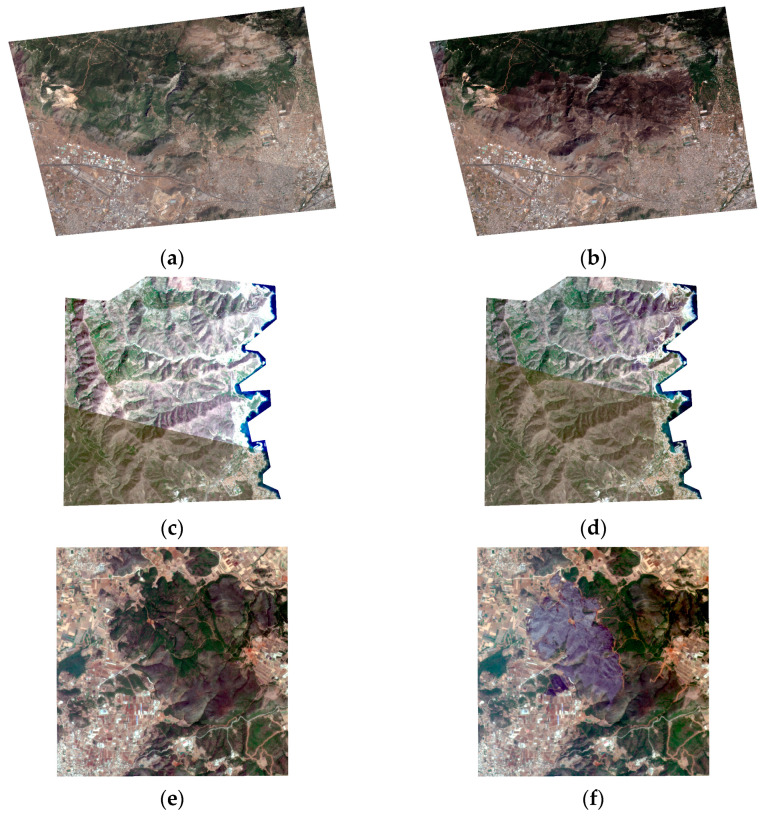

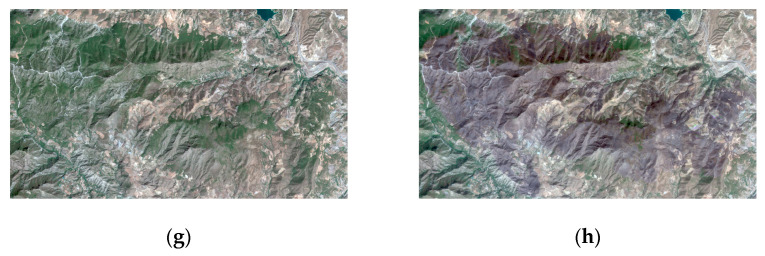
Pre-fire (first column) and post-fire (second column) images for the four selected events: (**a**,**b**) north Attica; (**c**,**d**) Portbou; (**e**,**f**) Euboea; (**g**,**h**) Sierra de los Guájares.

Figure 1 presents a side-by-side comparison of the pre-fire (first column) and post-fire (second column) images for the four distinct zones. Each row corresponds to a different location, illustrating the transformation of the landscape due to the fire event. As shown in Figure 1, comparing the pre- and post-fire images reveals radiometric differences between them. These differences are evident even within the image mosaic of the same day when acquired with different sensors (e.g., Figure 1c,d). Preprocessing for data harmonization could help balance the radiometric response of the images. However, to present a robust and automatic methodology and to test the characteristics of the SuperDove images, we decided not to perform this preprocessing. The effects of these radiometric differences are discussed in later sections. Also, Table 1 provides details of the image-capture dates for the four areas selected for the study.

To select the study area, the public geospatial information provided by the CEMS for each of the events was used.

### 2.2. Vegetation Index Selection

Since the SuperDove sensor does not have a SWIR band, it was necessary to determine the best vegetation indices to allow the detection of the burned areas. For this, nine vegetation indices were evaluated. For each one, the average difference between the pre- and post-fire images in the selected area was obtained. Prior to calculating this average value, images were filtered with the provided burned-area delimitation mask. The names, equations, and references of these nine vegetation indices appear in Table 2.

### 2.3. Methodology for Burn-Area and Severity Mapping

**Figure 2:**
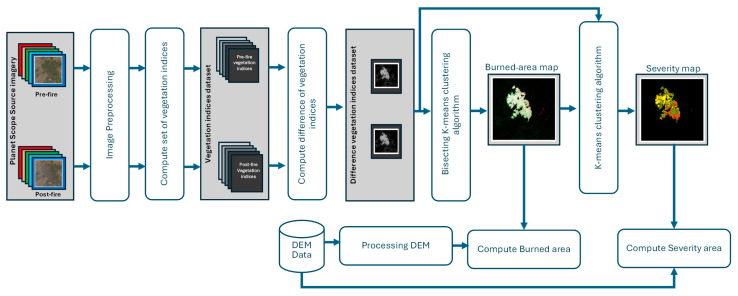
Scheme of the methodology to obtain the maps of burned area and fire severity.

Figure 2 shows the outline of the methodology applied to obtain the burned-area map, as well as the severity of the fire. First, the preprocessing of the time series is carried out. As already mentioned, the data provider already provides atmospherically corrected and orthorectified images. Therefore, at this stage, only the final mosaic of the pre- and post-fire images is carried out, as well as the adjustment of the digital levels. Next, the different vegetation indices are calculated for the pre- and post-fire images, as well as the differences between indices. As detailed in Section 3.1, an evaluation was conducted to assess the differences in each vegetation index before and after the fire. The objective was to identify the most appropriate indices for detecting burned areas and mapping fire severity. As a result of this evaluation, two indices were selected, and their pixel-by-pixel values were used as two-dimensional vectors for the clustering algorithm. To determine which pixels correspond to a burned area, bisecting K-means clustering is executed, and thus a standard threshold interval is avoided. Bisecting K-means is a clustering algorithm that is a hybrid between divisive hierarchical clustering and K-means clustering [51,52]. It employs a strategic iterative process to enhance precision. Initially, it considers the entire data set as a single cluster. This cluster is then divided into two distinct sub-clusters using the conventional K-means algorithm. Subsequently, the algorithm identifies the sub-cluster with the highest sum of squared errors (SSE) and selects it for further division. This iterative process of splitting continues until the algorithm achieves the predetermined number of clusters. By consistently bisecting the clusters and prioritizing those with the highest error, the bisecting K-means algorithm aims to boost the accuracy of clustering and ensure that data are grouped in a more meaningful manner. This algorithm provides some advantages over standard K-means. It provides a hierarchical structure to the clusters, which may be more informative than the flat clusters produced by standard K-means, and seems appropriate for the problem we address. Additionally, it is considered faster than normal K-means as it splits one group at a time, which can be more efficient. Finally, this method is particularly useful when dealing with large data sets and when a hierarchical cluster structure is desired. The two clusters correspond to burned and un-burned pixels.

Finally, a severity map is synthesized by running the K-means clustering algorithm [52], but only on the pixels filtered through the burned-area mask. Four classes are established as parameters of the algorithm, corresponding to each of the degrees of severity: low (insignificant to slight damage), low-to-moderate (moderately damaged), moderate to high (very damaged), and high severity (destroyed). Again, employing an automatic clustering algorithm avoids the use of fixed thresholds.

**Figure 3:**
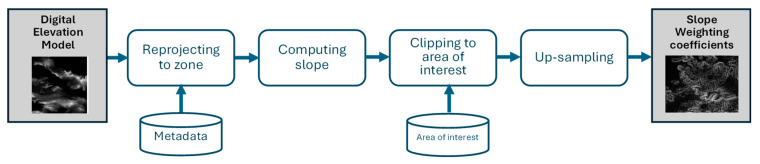
Processing of the DEM.

From both the burned-area map and the severity map obtained through the previous method, a pixel-by-pixel calculation of the areas can be performed. To obtain a more accurate measurement of the surface affected for each area, the surface is measured by its average slope extracted through a digital terrain elevation model (DEM). Figure 3 shows the DEM processing scheme to obtain the slope at each point that will be used as a weighting coefficient for each pixel.

Primary elevation data were obtained from the Shuttle Radar Topography Mission (SRTM), which flew aboard the space shuttle Endeavor from 11 February to 22 February 2000. This information was provided by the National Aeronautics and Space Administration (NASA) and the National Geospatial-Intelligence Agency (NGA) who participated in an international project to acquire radar data [53]. SRTM 1 Arc-Second Global elevation data (DOI number: /10.5066/F7PR7TFT) provide global coverage of gap-filled data with a resolution of 1 arc second (30 m). Moreover, this high-resolution global data set is distributed as an open source. The process (Figure 3) includes the reprojection of the area using the geographic information provided by the PlanetScope images as metadata. The slope of each point is then calculated and must finally be resampled to 3 m at the points of interest.

## 3. Results

The comprehensive analytical approach to testing the behavior of data provided by the SuperDove sensor, applied to the four fire zones, led to the following results, structured in three key components. Each of them contributes to establishing a reference framework for the use of these data in determining the burned area and the severity of forest fires, particularly small fires.

### 3.1. Selected Vegetation Index

The preliminary phase involves the collection and analysis of the data set for vegetation indices. These indices are likely to provide detailed information on the state of the land cover and, therefore, the difference between pre- and post-fire values could be used as an indicator of the state of the land cover. To test this, pixel-by-pixel differences were computed between the vegetation indices detailed in Table 2 for each pair of pre- and post-fire images. Finally, the average of the pixel values within the measured area was calculated using the fire delimitation vector mask provided by the CEMS.

**Figure 4:**
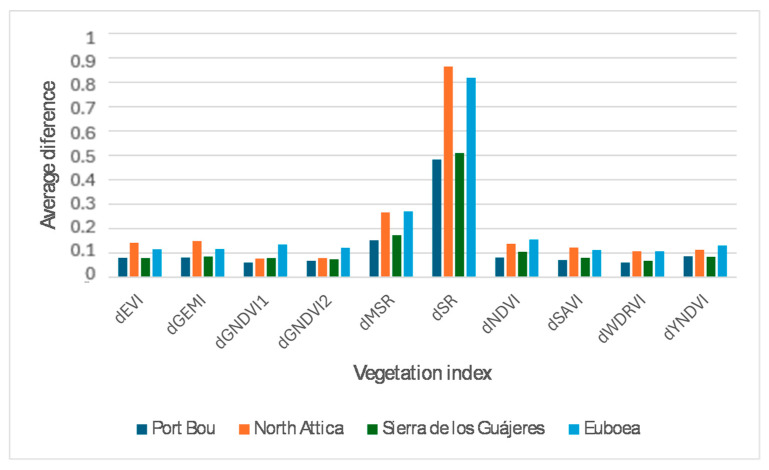
Average differences between vegetation indices in pre- and post-fire images.

Figure 4 shows the results of the average differences between vegetation indices in the images. At first glance, it seems to indicate that the greatest differences are obtained with the MSR and SR indices and that, therefore, these would be the ideal indices for delimiting burned areas.

**Figure 5:**
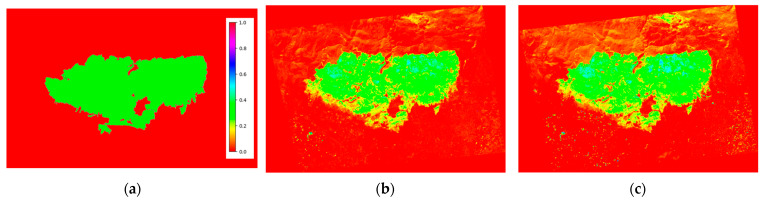

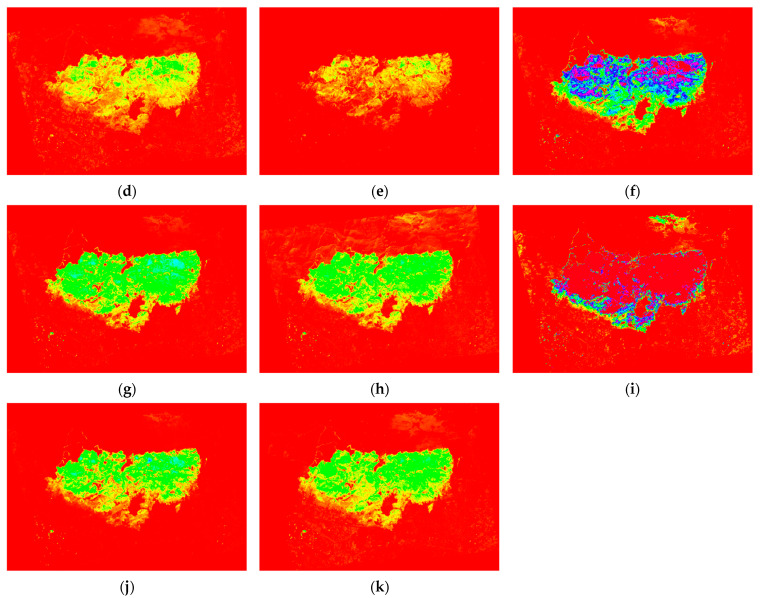
Difference between pre- and post-fire images for different vegetation indices in north Attica area: (**a**) fire delimitation mask provided by CEMS, (**b**) EVI, (**c**) GEMI, (**d**) GNDVI1, (**e**) GNDVI2, (**f**) MSR, (**g**) NDVI, (**h**) SAVI, (**i**) SR, (**j**) WDRVI, and (**k**) YNDVI.

However, if the pre- and post-fire differences in the different indices that appear in Figure 5 are observed in detail and, especially, if they are compared with the delimitation mask of the burned area provided by the CEMS (Figure 5a), the information presented the MSR and SR indices (Figure 5f and Figure 5i, respectively) makes the process of obtaining a burned-area mask difficult. This degraded performance is probably due to the definition of these indices (Table 2), in addition to the spectral responses involved in their calculation in the case of changes in vegetation status.

Similarly, a visual inspection of the variation in other vegetation indices, such as EVI, SAVI, and GEMI (Figure 5b, Figure 5e, and Figure 5h, respectively), allows the identification of various areas where changes in vegetation are detected that do not correspond to the area affected by the fire. This is likely due to the sensitivity of these indices to radiometric changes between the pre- and post-fire images. Although this aspect could possibly be corrected with a better preprocessing stage, these groups of indices were discarded because the aim was to achieve a robust and simple methodology for determining the burned-area extent and severity level.

Finally, there is a group of indices that seem to separate the burned area well from the rest of the land cover (if MSR and SR are excluded) and that are more immune to radiometric problems. Among them are the NDVI, WDRVI, and YNDVI (Figure 5g, Figure 5j, and Figure 5k, respectively). For the final automatic detection of burned areas, the first and last indices were used because they always appeared among those with the greatest average difference detected in the four study areas (see Figure 4). Although the inclusion of the WDRVI was tested in the following processing stages, the results did not vary substantially.

### 3.2. Burned-Area Maps

The results of the burned-area maps derived from Planet images using the described methodology contrast the data provided by CEMS reports. This comparison was carried out both qualitatively and quantitatively, through visual evaluation and surface measurement, respectively.

**Figure 6:**
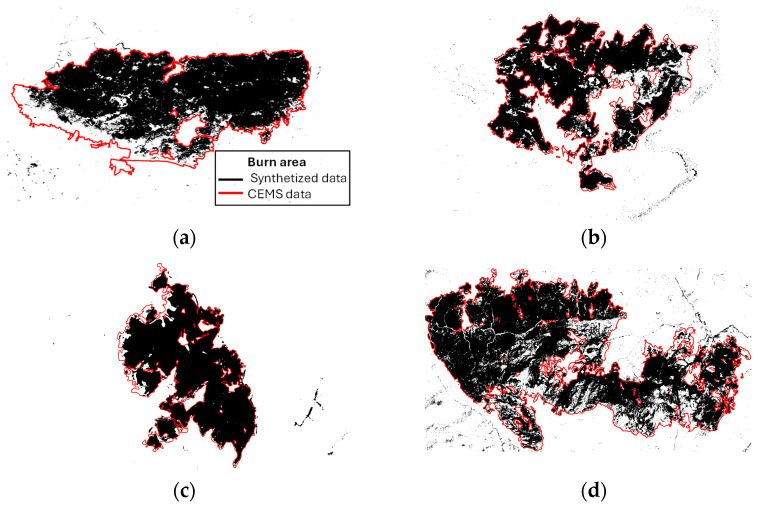
Comparative visualization of the burned areas computed using the described methodology (in black) and those extracted from the CEMS report (in red) for the fires in (**a**) north Attica, (**b**) Portbou, (**c**) Euobea, and (**d**) Sierra de los Guájeres.

Figure 6 presents the computed burned areas for the four distinct considered fire events. The inner section of each graph (in black) represents the computed burned areas using the automatic methodology described in Figure 2 and Figure 3. In the surrounding areas, depicted in red, it is possible to appreciate the boundaries of the burned areas obtained from the CEMS reports. This color-coded representation provides a comprehensive view of the extent of the fires and the additional areas identified by the CEMS. Except for the image in Figure 1a, the boundaries of the areas generated by the proposed methodology align well with those obtained from the geodata provided by the CEMS.

**Figure 7:**
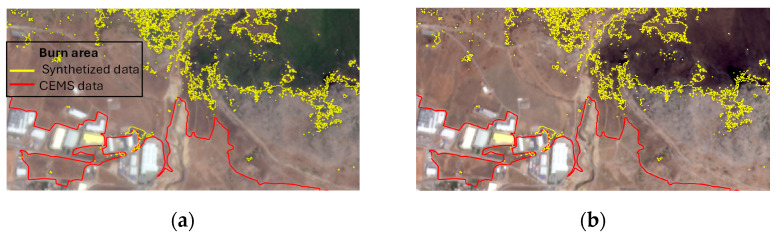
Detailed views of discrepancy in burned-area edges. The red line delineates the fire’s extent as per CEMS data, contrasting with the yellow line that maps the area using the specified methodology: (**a**) pre-fire, (**b**) post-fire.

In Figure 7, an enlarged view showing the details of the area for the north Attica fire, in which the edges of the burned areas do not coincide with the pre- and post-fire images, can be seen. The red line corresponds to the delimitation of the fire according to the CEMS reports, while the yellow line corresponds to the boundaries generated with the described methodology. The analysis of the images before and after the fire allows us to identify that, in most locations, the vegetation has not been burned and remains vigorous. However, it is true that there is a change in the bare ground that could be attributed to the fire.

A comparison between the estimation of the burned area (in hectares) and the area delimitation provided by CEMS reports appears in Table 3 for each study area. The applied methodology automatically classifies image pixels into two groups that correspond to the burned areas and untouched vegetation. Subsequently, the sum of the pixels labeled as “burned area”, weighted by the slope of each pixel’s central location, is calculated to obtain the total burned surface. However, the data provided by the CEMS refer to the delimitation of the burned area. This represents a different approach that involves reducing the number of objects based on their size [36] (p. 10), [37] (p. 12), [38] (p. 12), [39] (pp. 9–10). Consequently, although, as seen in Figure 6, the delimitation of the areas is reasonably similar (except in the case already mentioned in Figure 6a), the measurement of the surface affected by the fire may appear to be more different. In general, there is a trend towards overestimation in CEMS reports, which increases as the area affected by the fire increases.

### 3.3. Severity Maps

**Figure 8:**
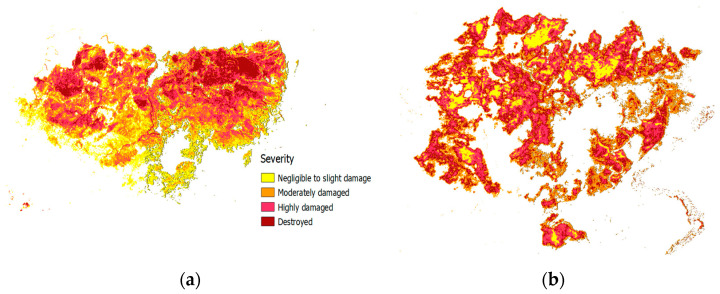

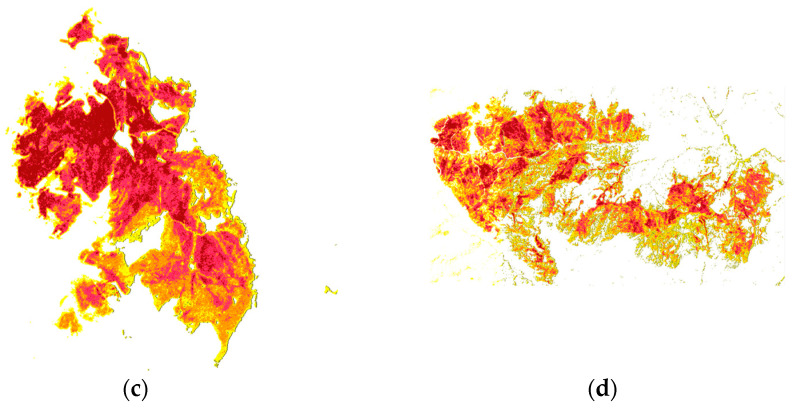
Synthetized severity maps computed using the proposed methodology: (**a**) north Attica, (**b**) Portbou, (**c**) Euobea, and (**d**) Sierra de los Guájeres.

Finally, Figure 8 and Table 4 show the severity maps for each of the study zones, as well as the quantitative data on the percentage of surface area over the total fire-affected zone for each severity class. Quantitative analysis by visual inspection shows the coherence of the severity maps obtained with the proposed methodology and those observed in the CEMS reports. However, the quantitative analysis shows a substantial discrepancy in the surface percentages assigned to each of the four categories used by the Copernicus Service: negligible to slightly damaged, moderately damaged, highly damaged, and destroyed.

To explain this discrepancy, the mechanisms of each approach should be considered. Indeed, the automatic approach proposed in this work uses a clustering algorithm that is inherently adaptive, while the procedure used in the CEMS reports is based on NDVI thresholding, for which the same thresholds are used in all scenarios. Indeed, if the percentages in Table 4 are analyzed, it is observed that there is a certain balance between the groupings generated by the proposed methodology and a lack of representativeness of degrees of severity in the CEMS data. For example, in the fire that occurred in north Attica, only 1% of the area was considered as destroyed. Unfortunately, establishing the validity of either one of these approaches requires field data, which is not available.

## 4. Discussion

This research tackled the critical challenge of accurately delimiting burned areas, particularly for smaller fires. This task becomes increasingly important as the alarming trend of global warming intensifies forest fires. By precisely mapping burned zones, including even small fires, it is possible to gain a deeper understanding of fire–climate relationships. This includes how longer fire seasons and drier conditions, fueled by rising temperatures, are impacting the extent and severity of fires globally. This information is vital for developing effective fire management strategies and mitigating the escalating consequences of global warming on world forests.

To address this point, this work explores the potential of vegetation indices derived from CubeSat imagery, focusing specifically on the SuperDove sensors in the PlanetScope constellation. The advantage of these sensors lies in their fast revisit time, which allows an almost daily monitoring of fires. However, challenges remain. Notably, the SuperDove sensor lacks an SWIR band, crucial for the commonly used NBR-based fire rating. Additionally, radiometric problems inherent to the sensor can introduce noise into the data, as noted in Section 2.1. Despite these limitations, the SuperDove constellation presents a valuable opportunity for fire monitoring and a chance to improve fire analysis, particularly in relation to the fast-moving dynamics of smaller fires.

To prove the validity of this assumption, a methodology was developed and applied to four recent fires in Greece and Spain that combined a series of characteristics, namely small fire size, areas of mountainous relief with orography pushing the functioning of vegetation indices to the limit, and the activation of the CEMS service, providing a report and geodata associated with each event. In these areas, the performance of nine different vegetation indices was compared for fire-extent mapping purposes. The results showed that the combination of the NDVI and the YNDVI provides accurate data about burned areas. These choices were motivated by their ability to detect significant average differences in burned areas in the four affected regions after a detailed quantitative and qualitative analysis. Finally, an automatic methodology based on hierarchical clustering algorithms was used to classify the pixels within the images. This methodology diverges from the classic classification approach to this type of problem based on fixed thresholds. In this sense, it appears that bisecting K-means clustering effectively separates pixels into burned and unburned categories, while K-means clustering provides greater granularity by delineating fire severity levels within burned areas. However, this last aspect requires additional research, the results of which must be compared with field data.

By combining frequent satellite imagery, informative vegetation indices, and powerful automatic clustering techniques, the proposed methodology offers a robust approach to the delineation of burned areas, especially for smaller fires, which is critical to understanding the evolution of fire–climate relationships in a warming world.

The burned-area maps synthesized with the proposed methodology largely reflect those prepared by the CEMS for three of the four studied areas. There was only one case where a slight difference arose. Similarly, fire-severity maps aligned well with the CEMS reports in identifying burned areas, although some variation emerged in the specific percentages of land categorized within each severity level. Additionally, with respect to the estimation of the burned area, it seems that the reports derived from the CEMS tend to overestimate the burned area. This discrepancy becomes more significant in larger fires. Also, this misalignment could be explained by the limitations of the proposed methodology, particularly when dealing with various fire-affected regions. Variations in soil type in these regions can significantly influence the spectral reflectance captured by the SuperDove sensor. Since the proposed method is based on vegetation indices, these variations in reflectance could lead to misinterpretations in burned-area calculations, especially for larger fires that span a broader range of soil types. Additionally, the radiometric noise inherent to the SuperDove sensor data could also contribute to these inaccuracies. More research is needed to explore alternative approaches or data-correction techniques that can take these limitations into account and improve the accuracy of burned-area delineation, particularly in regions with diverse soil compositions.

The fire-severity maps generated using the clustering algorithm identified burned zones that aligned with the Copernicus Emergency Management Service reports. However, there were significant discrepancies in the areas assigned to each severity level. This difference presumably arises from the different methodologies used. The proposed approach is based on a clustering algorithm that groups pixels into four predefined severity categories. In contrast, the CEMS uses established thresholds applied to the NDVI. While the automatic method offers flexibility, it may not be tailored as effectively to specific fire events compared to the standardized thresholds used by the CEMS. Unfortunately, the absence of field data in the study areas makes it difficult to definitively determine which approach is more accurate. Despite this limitation, the severity maps derived from the proposed methodology capture the general trends observed in the CEMS reports. To fully understand the discrepancies and validate the accuracy of each method, further research with ground validation data is necessary.

## 5. Conclusions

This study aimed to demonstrate the effectiveness of SuperDove imagery in wildfire detection and severity assessment. The results with the SuperDove images and the proposed methodology differed from the CEMS data on burned vegetation cover by less than 20%, considering the different approaches already described. The proposed methodology contributes to improving management strategies for such devastating events. In this sense, the following main conclusions can be listed regarding the methodology, sensor, vegetation indices, and fire mapping:

1. This study presents an automatic methodology to map burned areas and assess fire severity using images from the SuperDove sensor of the PlanetScope constellation. The methodology leverages vegetation indices and a combination of clustering algorithms to precisely delineate burned areas and classify them according to severity levels.
2. The results highlight the importance of appropriately selecting vegetation indices to accurately assess burned areas and wildfire severity. The indices that performed best in this study were NDVI and YNDVI, as they provided a clear distinction between burned areas and unaffected vegetation and were less susceptible to radiometric problems.
3. The SuperDove sensor, despite lacking an SWIR band, demonstrated effective mapping of burned areas when combined with specific vegetation indices and clustering algorithms.
4. Regarding severity mapping, the results of the SuperDove sensor in conjunction with this methodology, in contrast to the common approach provided by the CEMS, are not compelling and imply the need for future research exploring ground truth.
5. The methodology developed in this study enables a temporally detailed monitoring of wildfires and has potential to improve current fire management strategies. The results were compared with CEMS data, indicating their usefulness in various scenarios.

In summary, this work demonstrates the effectiveness of the images captured by the SuperDove sensor in the estimation of burned areas and severity levels, proposing an automatic methodology for mapping burned areas and fire severity.

## Figures and Tables

**Table 1:** Dates corresponding to the capture of pre- and post-fire images in the four selected areas.

Zone	Image Pre-Fire	Image Post-Fire	Number of Tiles
North Attica	20 August 2023	12 September 2023	2
Portbou	21 July 2023	7 August 2023	2
Euboea	20 August 2023	23 August 2023	1
Sierra de los Guájares	8 September 2022	5 October 2022	2

**Table 2:** Formulations and references for the different vegetation indices used in this work.

Name	Equation	Reference
Enhanced Vegetation Index (EVI)	$EVI=2.5*\frac{NIR-RED}{(NIR+6*RED-7.5*BLUE)+1}$	[42]
Global Environmental Monitoring Index (GEMI)	$GEMI=(n*\left(1-0.25*n\right)-\frac{RED-0.125}{1-RED}$), where $n=\frac{2*\left({NIR}^{2}-{RED}^{2}\right)+1.5*NIR+0.5*RED}{NIR+RED+0.5}$	[43]
Green Normalized Difference Vegetation Index (GNDVI)	$GNDVI=\frac{NIR-[540:570]}{NIR+[540:570]}$	[44]
Modified Simple Ratio (MSR)	$MSR=\frac{RDVI-1}{\sqrt{RDVI}+1}$, donde $RDVI=\frac{NIR}{RED}$	[45]
Normalized Difference Vegetation Index (NDVI)	$NDVI=\frac{NIR-RED}{NIR+RED}$	[46]
Simple Ratio (SR)	$SR=\frac{NIR}{RED}$	[47]
Soil-Adjusted Vegetation Index (SAVI)	$SAVI=\frac{1.5*\left(NIR-RED\right)}{NIR+RED+0.5}$	[48]
Wide Dynamic Range Vegetation Index (WDRVI)	$WDRVI=\frac{a*NIR-RED}{a*NIR+RED}$, where a goes from 0.1 to 0.2	[49]
Yellow Normalized Difference Vegetation Index (YNDVI)	$YNDVI=\frac{NIR-YELLOW}{NIR+YELLOW}$	[50]

**Table 3:** Comparison of burned-area estimation and CEMS area delimitation.

Zone	Estimated Burned Area	CEMS Area Delimitation Data	Difference
North Attica	4766.90	6076.21	1309.31 (21.55%)
Portbou	450.13	500.04	49.91 (9.98%)
Euboea	496.11	526.22	30.11 (5.72%)
Sierra de los Guájares	4131.95	5141.4	1009.45 (19.63%)

**Table 4:** Comparison of percentages of severity estimation between the proposed approach and CEMS report data.

Zone	Severity	Estimated Severity	CEMS Reports Severity
North Attica	Negligible to slight damage	25%	13%
	Moderately damaged	15%	48%
	Highly damaged	30%	38%
	Destroyed	30%	1%
Portbou	Negligible to slight damage	34%	83%
	Moderately damaged	32%	16%
	Highly damaged	24%	1%
	Destroyed	10%	0%
Euboea	Negligible to slight damage	28%	4%
	Moderately damaged	32%	0%
	Highly damaged	17%	49%
	Destroyed	23%	47%
Sierra de los Guájares	Negligible to slight damage	32%	12%
	Moderately damaged	31%	14%
	Highly damaged	25%	54%
	Destroyed	12%	20%

## Data Availability

PlanetScope imagery from Planets Lab PBC are used under the Education and Research Licensing of University of Pavia.
